# Case Report: Surgical treatment of type B aortic dissection in an adult with double aortic arch

**DOI:** 10.3389/fcvm.2024.1511677

**Published:** 2024-12-06

**Authors:** Chong Luo, Longrong Bian, Weitao Liang, Zhong Wu

**Affiliations:** Department of Cardiovascular Surgery, West China Hospital, Sichuan University, Chengdu, Sichuan, China

**Keywords:** double aortic arch, descending aortic dissection aneurysm, computed tomography angiography, esophagography, thoracic endovascular aortic repair (TEVAR)

## Abstract

**Background:**

Double aortic arch (DAA) with type B aortic dissection in adults is a rare aortic vascular disease. The abnormal anatomical structure of the aortic arch in such patients presents significant challenges in the selection of surgical approaches, and there is a notable lack of exploration into endovascular repair approaches that simultaneously preserve asymptomatic vascular rings.

**Case description:**

A 43-year-old female patient was admitted due to recurrent chest and back pain lasting for over a month. Computed tomography angiography (CTA) indicated a double aortic arch anomaly with localized dissection of the descending aorta. Esophagography with barium swallow revealed vascular indentation on the upper and middle thoracic esophagus, with mild to moderate local narrowing. Based on a comprehensive preoperative evaluation of the imaging and the patient's clinical history, a thoracic endovascular aortic repair (TEVAR) procedure was performed. Considering that the deformity did not cause any clinical symptoms and that the vessel diameter and distance from the proximal anchoring area were sufficient, the posterior section of the dominant arch was chosen as the proximal anchorage zone, and a stent with proximal bare zone was deployed to maintain blood flow to the distal non-dominant arch and preserve the integrity of the vascular ring. Follow-up CTA scans at one- and six-month post-operation showed that the aortic stent was well-positioned, with no visible primary lesion. The patient reported complete resolution of chest pain and no difficulties with swallowing or breathing.

**Conclusion:**

In adult patients with DAA complicated by aortic dissection, the abnormal anatomy of the aortic arch poses significant challenges in making treatment decisions. After a comprehensive, multidimensional evaluation of the patient's medical history, CTA, and esophagography, we successfully performed TEVAR procedure. This case provides new insights into the surgical strategy for treating such rare conditions.

## 1 Introduction

Double aortic arch (DAA) is a rare congenital cardiovascular anomaly, defined by the persistence of a right aortic arch in conjunction with the typical left aortic arch. The prevalence of the condition is estimated to be between 1% and 2% of all congenital cardiovascular malformations (1). As a result of compression of the esophagus and trachea by the vascular ring formed by the double aortic arch, children frequently present with symptoms such as dysphagia, vomiting, wheezing, and dyspnea. In cases where symptoms are evident, prompt surgical intervention is recommended (2). Conversely, for patients exhibiting only mild esophageal or tracheal compression without overt symptoms, a conservative approach may be appropriate. However, the optimal surgical strategy for asymptomatic cases of DAA in conjunction with type B aortic dissection in adult patients remains uncertain.

## 2 Case presentation

**Figure 1:**
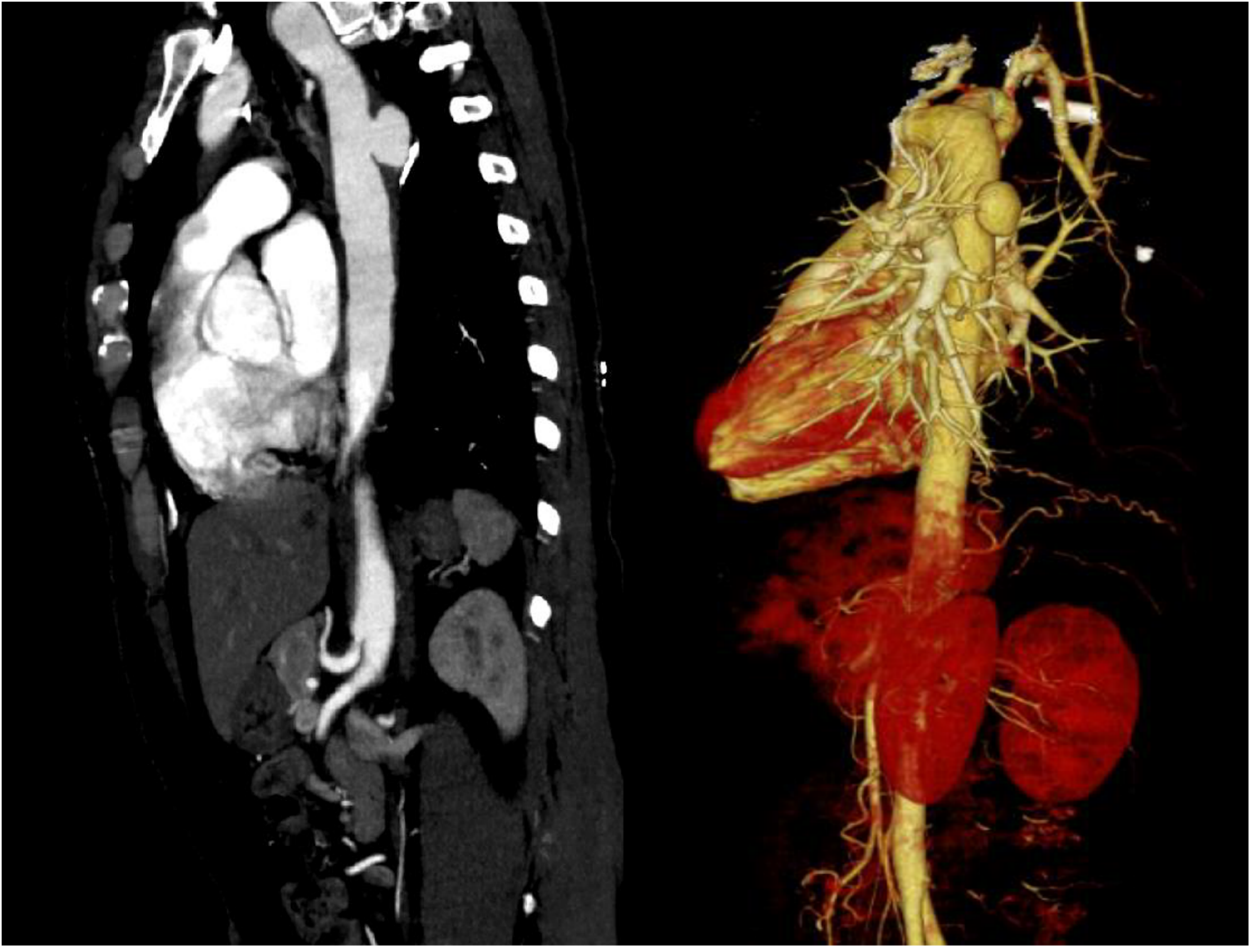
CTA indicated that aortic double arch malformation with suspected development of dissection in the descending aorta.

**Figure 2:**
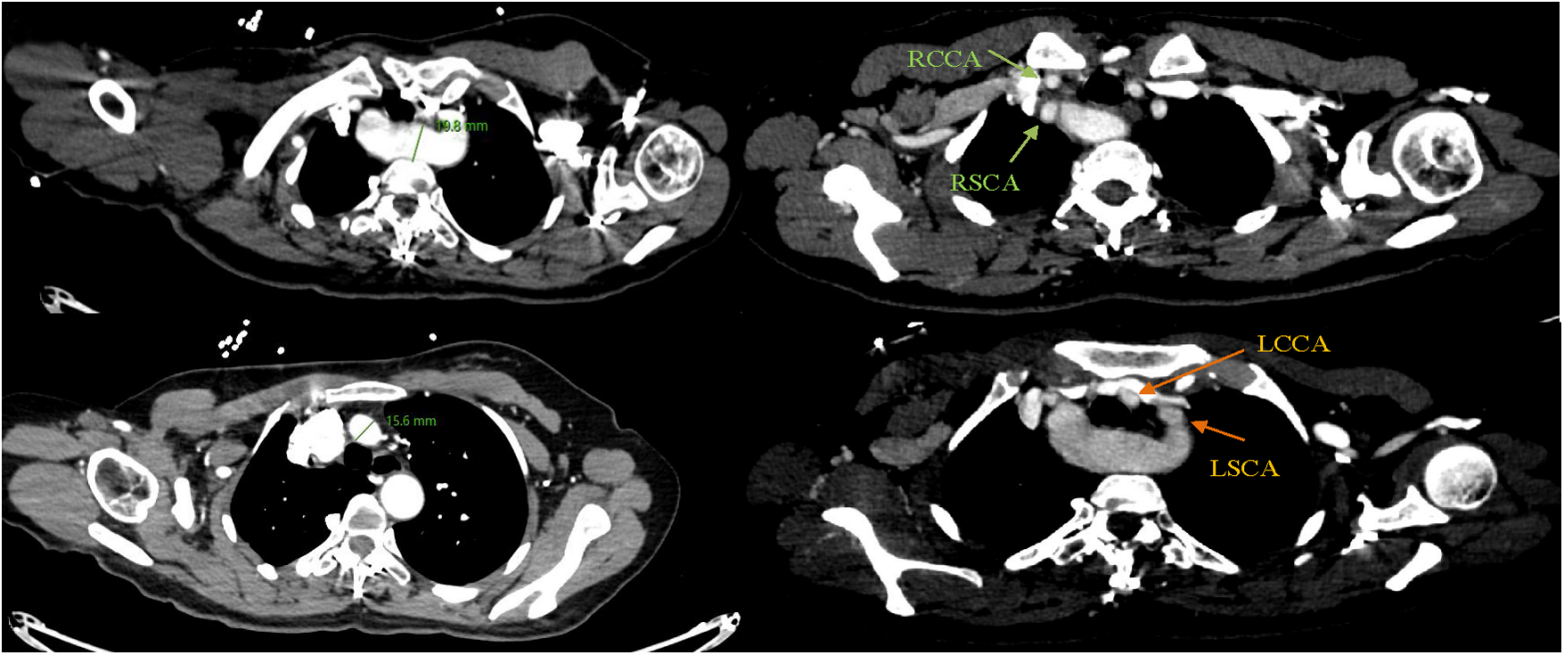
Right common carotid artery (RCCA) and right subclavian artery (RSCA) were originated from dominant right aortic arch, left subclavian artery (LSCA) and left common carotid artery (LCCA) were originated from left non-dominant aortic arch.

A 43-year-old female patient was admitted to the hospital due to recurrent chest and back pain for more than a month. CTA report indicated: aortic double arch malformation with dissection aneurysm in the descending aorta (Figure 1), right common carotid artery and right subclavian artery were originated from dominant right aortic arch, left subclavian artery and left common carotid artery were originated from left non-dominant left aortic arch (Figure 2).

**Figure 3:**
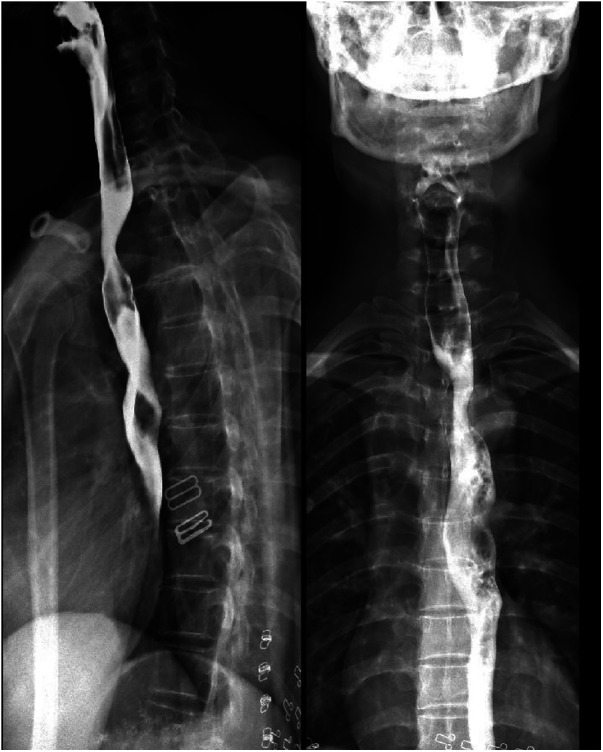
Barium meal examination of the esophagus suggested vascular indentation in the upper-middle segment of the esophagus, with mild-moderate narrowing.

The patient herself complained of no respiratory or swallowing problems. A barium meal examination of the esophagus indicated the presence of vascular indentation in the upper-middle segment, accompanied by local mild-to-moderate narrowing (Figure 3).

**Figure 4:**
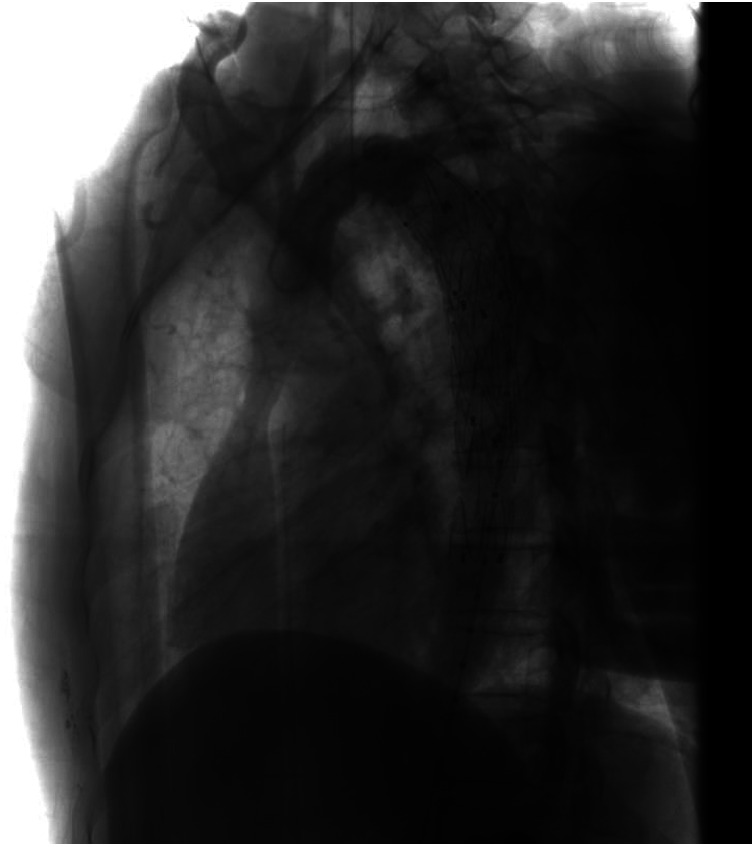
A TEVAR procedure was performed. The posterior section of the dominant arch was chosen as the proximal anchorage zone, and a stent with proximal bare zone was deployed to maintain blood flow to the distal non-dominant arch and preserve the integrity of the vascular ring.

**Figure 5:**
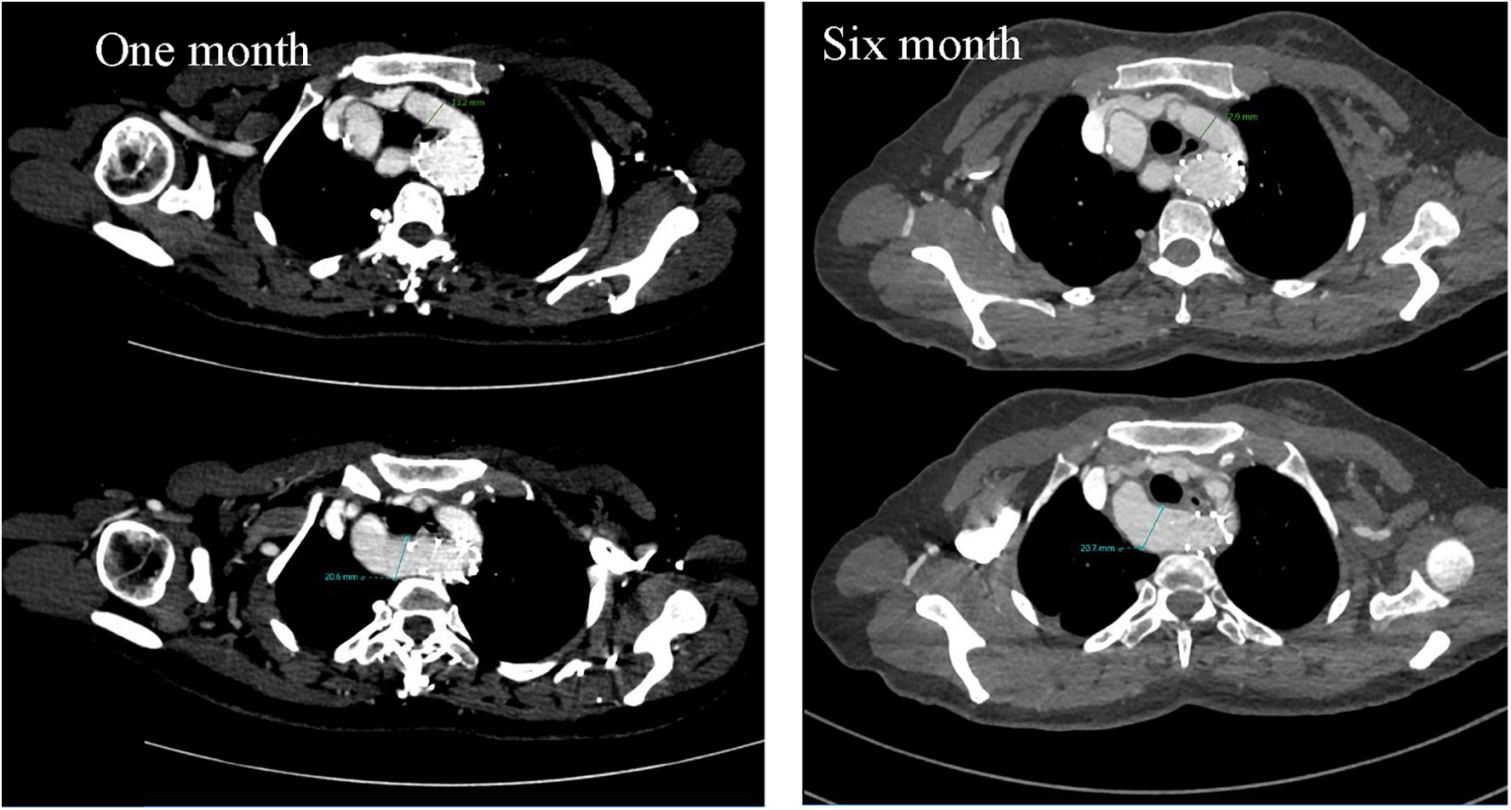
Cross-sectional images of postoperative CTA at one month and six months suggested no significant changes in dominant and non-dominant arch diameters and no obvious change as preoperatively.

**Figure 6:**
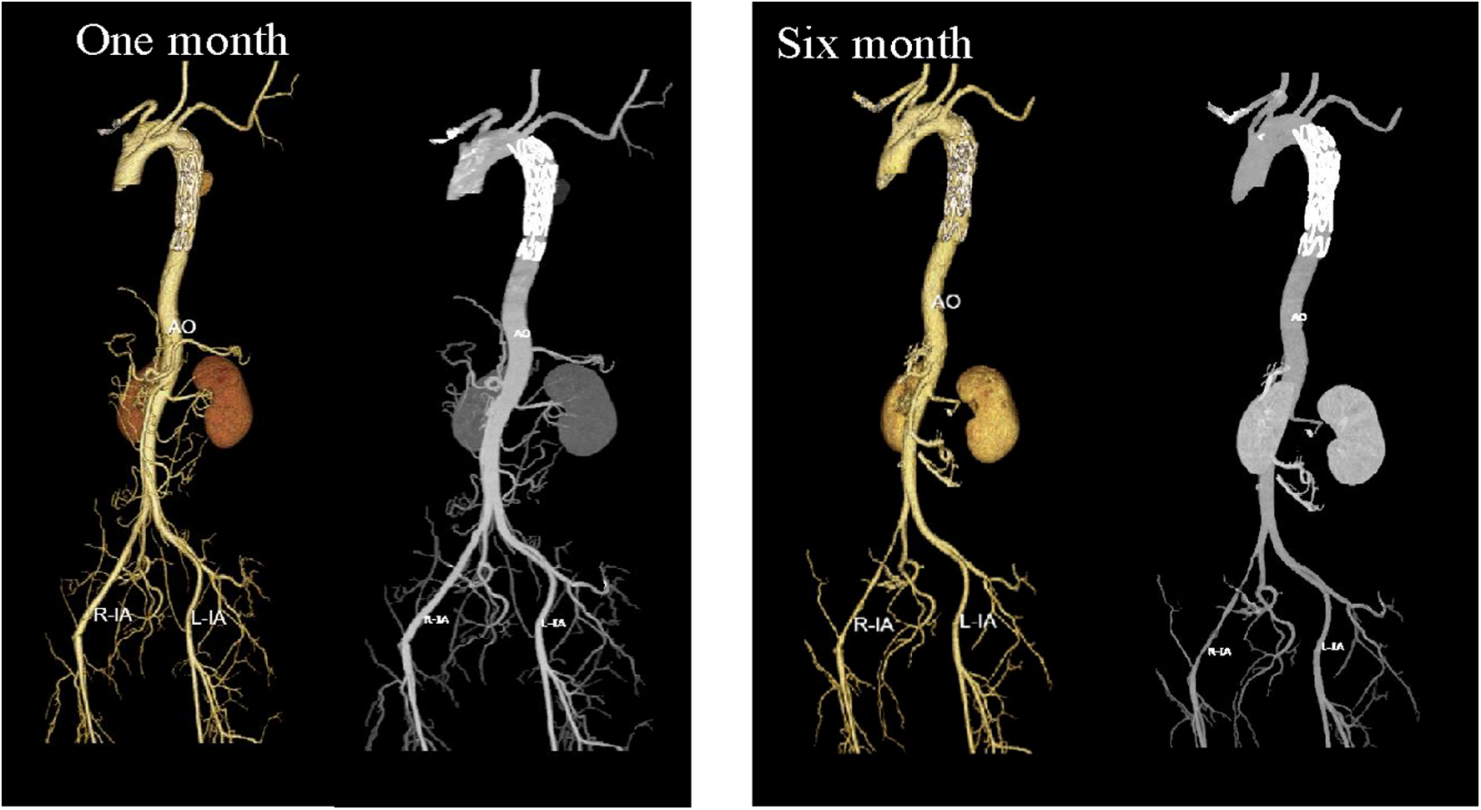
Three-dimensional reconstruction images of postoperative CTA at one month and six months suggested good stent positioning, with no visible evidence of the primary lesion.

A TEVAR procedure was performed. Considering that the deformity did not cause any clinical symptoms and that the vessel diameter and distance from the proximal anchoring area were sufficient, we decided to anchor the proximal bare stent area to the posterior portion of the dominant arch, and at the meantime, the bare region would not obstruct the non-dominant arch flow, preserving the original aortic arch blood flow (Figure 4). Follow-up CTA scans at one- and six-month post-operation showed that the aortic stent was well-positioned, with no visible primary lesion. The patient reported complete resolution of chest pain and no difficulties with swallowing or breathing (Figures 5, 6).

## 3 Discussion

DAA could be categorized as follows: (1) right arch dominant, 75%; (2) left arch dominant, 15%; (3) double arch balanced, 10% (3), and was often characterized by symptoms of tracheal compression, such as coughing and wheezing. Prolonged compression led to tracheal achondroplasia, which significantly affected growth and development, and even caused mortality. The DAA's disease management process has been well established, asymptomatic or mildly symptomatic children were operated on at 6–9 months of age, and symptomatic children were selected for surgery after a CT scan at 2–4 months of age (4). Very few people with DAA would grow into adulthood asymptomatic and be identified because of paroxysms of breathlessness, dysphagia, and other discomforts (5–7). Aortic dissection has commonly been described as a sudden onset of severe, tearing chest pain, and studies have found that hereditary thoracic aortic disorders (HTAD), such as Loeys-Dietz syndrome, Marfan's syndrome, and bicuspid aortic valves, have been associated with the incidence of aortic dissection (8). However, there has not yet been sufficient confirmation of an association between DAA with HTAD and aortic dissection.

The decision-making for treatment strategies could be complicated when congenital and acquired diseases occur in close structural proximity, and the challenge for DAA combined with type B aortic dissection was the abnormal arch malformation. Surgical treatment of related cases is rarely reported in the literature. Midulla (9) and Min (10) performed thoracotomy on three patients with DAA complicated by type B aortic dissection, transecting the non-dominant arch to relieve tracheal and esophageal compression from the vascular ring and performing graft replacement. However, intraoperative complexities included prolonged dissection and reconstruction times. With advancements in endovascular devices and techniques, TEVAR has become highly customizable and minimally invasive is now widely applied in the treatment of various complex type B aortic dissections (11, 12). In patients with DAA combined with type B aortic dissection, when there is no clinical or imaging evidence of significant tracheal or esophageal compression, preserving and reconstructing the original vascular ring structure while addressing only the dissection may offer a simplified yet effective approach. Zhao et al. (13) performed a TEVAR procedure on a patient with DAA and type B aortic dissection, reconstructing the right common carotid artery, left common carotid artery, dominant arch, and non-dominant arch. As we thought that the characteristic “Oversize” feature of the stent might exacerbate vascular ring compression. Consequently, the posterior section of the dominant arch was chosen as the proximal anchorage zone, and a stent with proximal bare zone was deployed to maintain blood flow to the distal non-dominant arch and preserve the integrity of the vascular ring. Follow-up CTA results at one and six months postoperatively demonstrated complete exclusion of the aortic dissection without significant changes to the vascular ring structure.

## 4 Conclusion

The TEVAR procedure appears to be an effective and less invasive treatment for patients with DAA complicated by type B aortic dissection. However, preoperative evaluation is crucial to assess whether there is a mismatch between the proximal and distal diameters of the stent graft due to aortic arch malformation. Additionally, it is important to consider the potential for increased esophageal or tracheal compression after stent graft placement in the aortic arch due to oversizing, as well as the possibility of the stent blocking the non-dominant arch.

## Data Availability

The original contributions presented in the study are included in the article/Supplementary Material, further inquiries can be directed to the corresponding author.
